# Nursing in the United States

**Published:** 1916-05-06

**Authors:** 


### THE MATRONS' AND SISTERS' DEPARTMENT.

### NURSING IN THE UNITED STATES.

The incorporation of the College of Nursing must quicken interest in the nursing methods and training-school organisations and societies in the United States. We have, therefore, pleasure in calling attention to the April number of the *American Journal of Nursing*, which is the foremost and most advanced journal of nursing in America. The number referred to would appear to be a special one, and contains 114 pages of closely printed matter. The articles on the Training School Record in After Years, Plans for the Reorganisation of the American Nurses' Association, The Educational Function of the Hospital, The Department of Nursing Education, Nursing News and Announcements, and The Official Directory, with others, may prove helpful at this time to those matrons devoting their thoughts to the organisation of the College of Nursing. Most of these have a practical value, as indeed has the whole April number. It is packed with information of interest to students of American nursing methods, but, being mainly, we believe, a business number, it has not perhaps equal interest to the general reader to that of an ordinary issue of this excellent monthly. Copies may be obtained from The Scientific Press, Limited, 28 Southampton Street, Strand, London, W.C.

